# Addressing neglected tropical diseases in Africa: a health equity perspective

**DOI:** 10.1186/s41256-023-00314-1

**Published:** 2023-07-25

**Authors:** Nsikakabasi Samuel George, Success Chekwube David, Maxencia Nabiryo, Blessing Abai Sunday, Omotayo Faith Olanrewaju, Yonah Yangaza, Deborah Oluwaseun Shomuyiwa

**Affiliations:** 1 Kano State Ministry of Health, Kano, Nigeria; 2 Faculty of Pharmaceutical Sciences, University of Nigeria, Nsukka, Nigeria; 3 Integrated Community Health Initiative Organization, Kampala, Uganda; 4 School of Public Health, Department of Community Health and Behavioural Sciences, Makerere University College of Health Sciences, Kampala, Uganda; 5 Faculty of Pharmacy, University of Uyo, Uyo, Nigeria; 6 Muhimbi University of Heath and Allied Science, Dar es Salaam, Tanzania; 7 Faculty of Pharmacy, University of Lagos, Lagos, Nigeria

**Keywords:** Neglected tropical diseases, Health equity, Africa, Epidemiology, Social determinants of health

## Abstract

Africa accounts for over one-third of the global burden of neglected tropical diseases (NTDs). Although continental efforts have been made to combat these diseases, there still exists a significant gap in the fight, ranging from a lack of data to multisectoral participation and, most critically, health inequity. Here, we assess the effort made to combat challenges caused by health disparities to prevent and control neglected tropical diseases. This article engages a health equity view to addressing the need for inclusion in achieving universal health coverage towards eradicating NTDs and outlines strategies to achieve such. Health disparities exist, and there is substantial and irrefutable evidence for them. Inequitable distribution and limited access to basic and essential life resources such as water, housing, toilets, soap, and literacy continue to facilitate the existence of NTDs such as Schistosomiasis, soil-transmitted helminths, and trachoma, the occurrence of which can be avoided if affected populations have better access to those resources. To eradicate NTDs, health disparities must be addressed to provide excellent health care to all populations and adequate universal health coverage for long-term sustainability. NTD programmes need to be data-driven to ensure better decision-making and ensure the inclusion of diverse population groups including women, children, and youths. This will ensure that no one is left behind, drawing upon the sustainable development goals. Community participation and engagement should also be considered as an essential approach to ensure people are at the centre of health programmes and their implementation.

## Background

Neglected tropical diseases (NTDs) are a collection of 20 chronic conditions that significantly affect the poor and vulnerable in tropical and subtropical areas [1, 2]. These diseases have been found to have affected more than one billion people, with differences in prevalence across the 47 member states of the World Health Organization (WHO) African Region [2]. In Africa, particularly the Sub-Saharan region, its prevalence is recorded in about half a billion of its population, with varied proportions amongst age, gender, etc., resulting in inequity [1]. The majority of NTDs are caused by a virus, bacterium, protozoon, or helminth. The phrase "neglected" connotes that these diseases have lately been rediscovered after being eclipsed for many years by other diseases such as HIV, tuberculosis, and malaria [3]. Schistosomiasis, Lymphatic filariasis (Elephantiasis) and Onchocerciasis (River blindness) have the highest prevalence globally, with 192 million cases, 51 million and 37 million cases, respectively [1]. Others are: Buruli ulcer, Chagas disease, dengue, echinococcosis, foodborne trematodiasis, human African trypanosomiasis (sleeping sickness), leishmaniasis, leprosy, lympha, rabies, soil-transmitted helminthiasis (intestinal worms), taeniasis/cysticercosis (pork tapeworm), blinding trachoma, and yaw, as listed by the WHO [2].

From the lenses of the Sustainable Development Goals (SDGs) agenda which aims to close the gap between marginalized populations' disproportionate health burdens and outcomes, NTDs further exacerbates health inequity [4]. NTDs are associated with disabilities and cognitive impairment in children, limiting their access to a good education and proper development [5]. Apart from the devastating health consequences, there are also socioeconomic consequences, which arise from the multiplicative effect on population productivity and a lack of access to quality healthcare [5, 6]. Factors such as marginalization, poverty of the affected population, a lack of funding for healthcare systems, and inadequate data on disease prevalence and control have all contributed to the diseases' neglect, making them a public health challenge [7, 8]. So far, control and elimination efforts have focused on cost-effective interventions [9]. With a stronger focus on prevention, basic NTD control strategies include vector control, improved hygiene, and a higher standard of living.

## Prevalence and distribution of NTDs in Africa

**Fig. 1:**
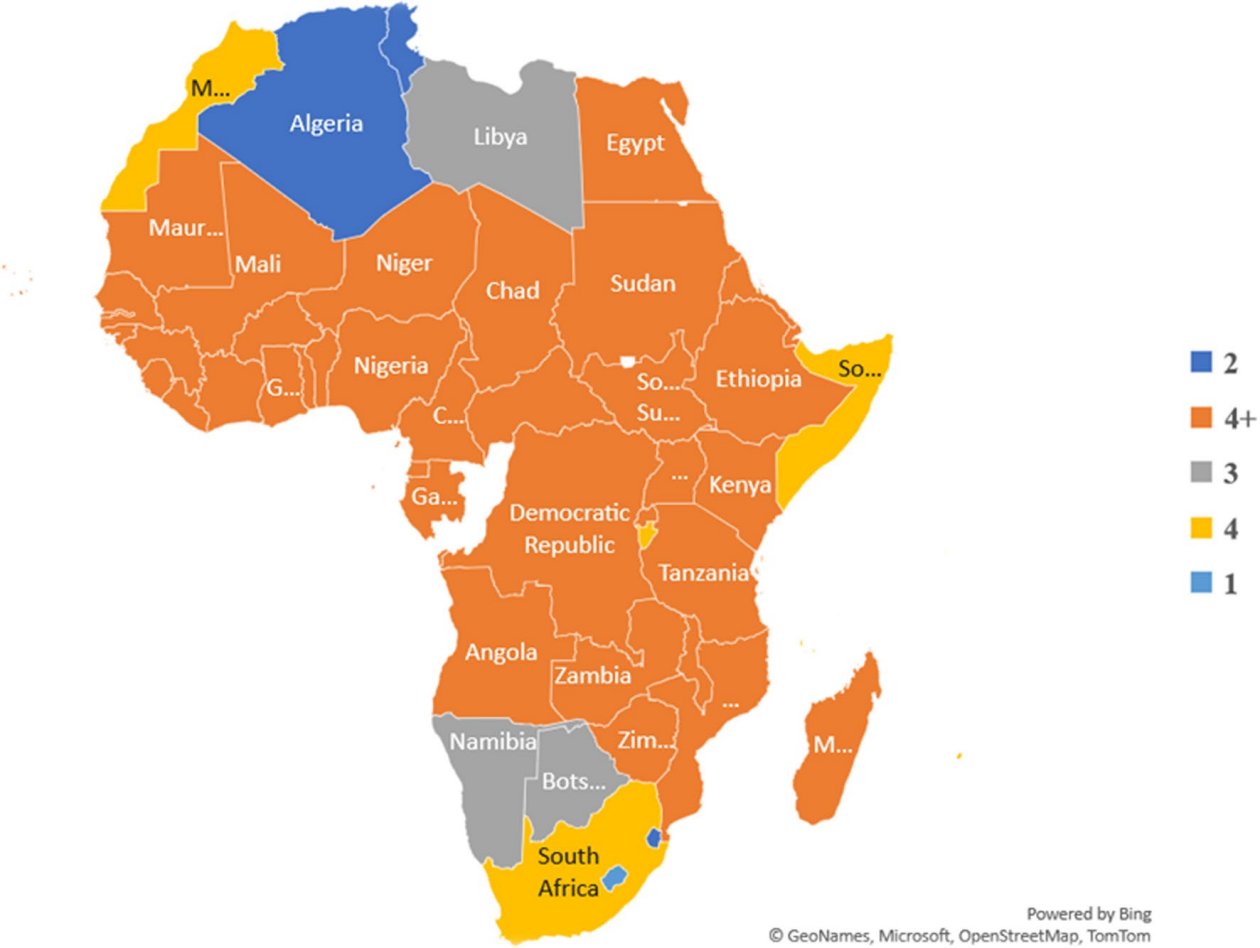
NTDs distribution among African countries

Africa accounts for about 40% of the global burden of NTDs, with about 600 million individuals requiring treatment [10, 11]. While NTDs are known to parts of the global community, at least one NTD is endemic in every country in the African Region, and 79% of African countries are co-endemic, with at least five of them [12, 13]. At least four NTDs have been identified in 47 African countries, spanning West Africa, East Africa, Central Africa, and parts of North and Southern Africa (see Fig. 1).

Schistosomiasis is a major public health problem in Africa, especially in Sub-Saharan Africa [14]. With an estimated 112 million people infected and an estimated 800 million people at risk of infection, *S. haematobium* is the most common species in Sub-Saharan Africa [14, 15]. Although Sub-Saharan Africa has just about a third of the world's population, it accounts for up to 90% of schistosomiasis cases, with an estimated 280,000 schistosomiasis deaths yearly. The world's highest schistosomiasis prevalence is currently found in Nigeria and West Africa.

Onchocerciasis affects 37 million individuals, with nearly all of those affected living in 31 African nations [13, 16]. Lymphatic filariasis is a preventable NTD endemic in Sub-Saharan Africa. In 2018, the estimated at-risk population in Africa requiring intervention was 341 million people [17]. The prevalence of soil-transmitted helminths in sub-Saharan Africa in children aged 5–14 years is 13% [18].

In total, 44 African countries have a widespread occurrence of at least one preventive chemotherapy (PC) responsive NTD, with 42 having at least two [11]. Populations at risk for preventative treatment in the WHO African Region range from 123 million for onchocerciasis to 470 million for lymphatic filariasis. Buruli ulcer, human African trypanosomiasis, and leprosy are the most common case management NTDs, with 3443 cases, 7197 cases, and 25,231 cases, respectively [13]. PC can cure and prevent five of these NTDs, thus reducing their burdens [11, 12].

## Efforts to combat NTDs

Investment in assessing the NTDs burden has played a crucial role in evaluating the trends and effectiveness of existing interventions, stratification of interventions, and validating the need to set improved strategic actions to beat NTDs through innovation, research and technological advancement [19]. Through drug discovery, diagnostics, vector surveillance, data management, digital mapping, technological advancements and scientific innovations, the NTD fight has drastically changed. There has been significant improvement in case prevention, detection, management, and treatment, reducing the burden of NTDs over time [5].

Africa has seen accelerated international efforts to eradicate NTDs over the last decade, with coordinated efforts from governments, health and development groups, donors, and commercial firms. These efforts culminated in the London Declaration of January 2012, when parties pledged to regulate or eliminate 10 NTDs by 2020 [20]. The Expanded Special Project for the Elimination of Neglected Tropical Diseases (ESPEN) was established by the World Health Organization's Regional Office for Africa in May 2016 to coordinate actions by NTD-affected countries, as well as public and private entities, to accelerate the elimination of NTDs in Africa by 2020. In the fight against NTDs, the ESPEN Partnership has made considerate progress [11, 21]. Togo declared lymphatic filariasis a public health hazard in 2017, while Ghana declared trachoma a public health problem in May 2018. Kenya became the 41st country in the African Region to be certified Guinea worm disease-free in February 2018, out of 47 countries. Leprosy is being phased out as a public health issue, and Human African Trypanosomiasis is also on its way out. The ESPEN's crucial role in the fight against NTDs through multisectoral partnerships achieved outstanding successes drawing on the London Declaration.

Every year on January 30, World NTD Day serves as a catalyst for turning awareness into action, securing additional resources for NTDs, and, most importantly, facilitating political leadership and ownership of NTD programs from afflicted nations. In 2021, this coincided with the launch of the NTD road map for 2021–2030 on January 28 2021 [22]. The road map lays out global goals and milestones for preventing, controlling, eliminating, or eradicating 20 diseases and disease groupings, as well as cross-cutting targets associated with the Sustainable Development Goals. The global aims for 2030 are to reduce the number of persons requiring NTD treatment by 90%, eliminate at least one NTD in 100 countries, eradicate two illnesses (dracunculiasis and yaws), and reduce disability-adjusted living years (DALYs) associated with NTDs by 75% [22, 23]. In a Memorandum of Understanding with the Uniting to Combat NTDs signed on March 29, 2022, the African Union pledged to end NTDs across all AU member states by 2030, highlighting commitments to strengthen collaboration and cooperation among stakeholders to control and eliminate twenty NTDs, achieving the vision outlined in the "Agenda 2063 'The Africa We Want'" framework [24].

## Health equity and relevant issues in Africa

According to the WHO, health equity is "the absence of unfair, avoidable, or remediable differences among groups of people, whether those groups are defined socially, economically, demographically, or geographically, or by other dimensions of inequality such as sex, gender, ethnicity, disability, or sexual orientation" [25]. Social determinants of health are circumstances in which people are born, grow, live and work that influence health [25]. They encompass economic factors, social factors, political, economic factors, environment, etc. Social determinants strongly influence health and health equity as they can create a background for health disparities [25, 26].

Every person should be able to achieve optimal health without discrimination based on ethnic group, race, religion, or other affiliation. A nation's prosperity depends on its population's health; a healthy population culminates into productive workers and increased revenue for the country through active service [26, 27]. Health equity comprises more than just health distribution and healthcare provision; it also encompasses broader issues of justice and fairness in social institutions [28, 29].

Infectious and parasitic diseases continue to be the leading cause of death in low- and middle-income nations, particularly in Africa, and the epidemiological transition is slowly taking place [30]. In remote areas across Africa, infrastructures such as healthcare facilities are lacking, and where they are present, skilled healthcare workers are scarce and typically concentrated in large cities [31]. The socioeconomic, cultural, political, ethnic and racial factors that influence the difference in health outcomes in Africa contribute to the poor-rich health gap between and within the African region.

Many people at the bottom of the socioeconomic ladder have limited options and opportunities to avoid being unwell and avoiding long-term disability and premature death linked with bad health conditions [29]. The gap between the rich and the poor in Africa lingers, and various factors such as education, gender, and poverty play a massive role in health disparities [26, 30]. Poverty severely impacts families, limiting access to health care services and increasing out-of-pocket costs, pushing families deeper below the poverty line [32, 33]. Because most national health insurance plans are ineffective, catastrophic medical expenses are unavoidable [34, 35]. This is a major concern because why should a person suffer to achieve excellent health, and how can public health be properly practised if the necessary resources are only available to the wealthy? As a result, increasing health equity remains a top priority for efficient and successful public health practice that cannot be overstated [36].

Ethnic factors in Africa also play a role in health inequity as indigenous populations, mostly pastoralists and nomads, who reside in hard-to-reach areas, have little or no access to health care. Hence, high morbidity and mortality are recorded among this group [37].

Furthermore, the right to equity of health and non-discrimination is critical to realizing the right to health within the international human rights framework [29]. Neglected Tropical Diseases (NTDs) are still prevalent in many parts of Sub-Saharan Africa that have been left behind by socioeconomic development. As a result, these diseases are indicators of extreme poverty and unfairness, exacerbated by the political, economic, social, and cultural institutions that influence health and wellbeing. This shows an urgent need to address such vulnerable groups' needs to achieve Sustainable Development Goals [38].

Health inequity across Africa shows that a thorough and systematic approach is needed to address the challenge so that no one is impeded from realizing their full potential because of their social status or other socially determined situations. Is achieving health equity possible in Africa? This is a startling question that one must examine because it appears to be impossible.

## The need for health equity and inclusion to eradicate NTDs

Most NTDs’ epidemiology reflects various health inequities as drivers of the NTD burden. In developing countries, NTDs are more common than in developed countries [1, 3]. NTDs disproportionately affect the marginalized populations living in poverty and dire environmental and social conditions, which facilitate the occurrence of many NTDs in developing nations' mainly constrained economies and struggling healthcare systems. Inequitable distribution and limited access to basic and essential life resources such as water, housing, toilets, soap, and literacy continue to facilitate the existence of NTDs such as Schistosomiasis, soil-transmitted helminths, and trachoma, the occurrence of which can be avoided if affected populations have better access to those resources [39, 40]. While several effective interventions have been/are being developed, such as drugs for treating NTDs, their full effectiveness will be realized only when health barriers such as poverty, poor housing, and poor sanitation are addressed [19, 41].

Existing research indicates that interventions developed through the lens of human rights are effective in changing conditions that exacerbate diseases, promoting improved service delivery and health equity. Poverty and inequity are considered to be both indications of NTDs. Thus, its prevalence shows the extent to which universal health provision has or has not been made available to endemic regions [2, 5]. This inequity extends to inadequate community engagement and poor healthcare offerings. According to an NTD programme carried out in Cameroon for the mass distribution of drugs against Schistosomiasis, it was observed that there was an inequity in achieving coverage for the population. Analysis showed repeated exclusion of some groups, such as indigenous and migrant farmers, women of reproductive age and school-age children from the MDA campaign [42]. The barriers to participation included poor and insufficient sensitization processes, limited community drug distributors, training and capacity, inequitable distribution and lack of alignment between distribution and availability of many population groups [42]. These are important barriers to healthcare delivery in endemic areas and result in poor diagnostics and treatment. The lack of healthcare knowledge within communities is depicted by the paucity of awareness of different aspects of diseases, including possible symptoms, transmission, prevention and outcomes [42].

Additionally, geographical location also serves as a barrier, as accessing healthcare during emergencies can be challenging [1]. Thus, medical services should be improved and integrated into such areas to reduce patients' journeys to access healthcare. Most importantly, finance has remained one of the utmost aspects of accessing healthcare since some medicines are not provided for free, which entails paying out of pocket to acquire them [35]. Despite that, it is notable that Tanzania has made progress in combat against NTDs, in addition to 46 countries that have eradicated at least one NTD. A systematic study by Banda et al. showed that adequate funding, community participation and good governance are pivotal to the successful integration of the NTD programme, which is in conjunction with the Kigali Declaration of NTDs earlier this year [43] while suggesting that government should incorporate NTDs in the national health plans and budget for accountability an ensuring sufficient financing [44].

Health equity inclusion supports improved health governance and leadership, allowing health systems to reflect better on the populations they serve while reducing health inequities. Health equity inclusion assures everyone, including the last mile, has access to NTD prevention services. In addition, another study carried out in Nigeria to optimize the performance of frontline implementers engaged in the NTD programme in order to strengthen community health to achieve universal health coverage highlighted the need to include NTD implementers in programme planning, review or research agenda to enhance their knowledge and acquire the necessary skills needed for efficient and effective service delivery [45]. As a result, making NTD preventive services available and essential in primary healthcare settings will go a long way toward achieving this goal. To eradicate NTDs, human rights-based programs that address health disparities among affected populations must be developed and promoted [41]. Achieving a sustainable reduction in NTD mortality requires deploying a multisectoral and multidisciplinary collaboration for health promotion, disease prevention and strengthening service delivery through primary healthcare, especially in underdeveloped populations [46, 47].

## Recommendations

Community Participation, including the integration of formal and informal social networks, should be a mainstay. NTD projects can utilize existing knowledge of marginalized populations and community perceptions and principles to coordinate programs. Local governance structures are essential to develop trust and drive sustainable programs. Collaboration with local stakeholders will help to improve health-related messaging and contribute to community-level monitoring of activities and behaviours. People from governance structures, community health workers, local authorities such as district heads, councils, and local health centres, as well as people with local knowledge, insights, and strengths, will likely improve partnerships and solution development, as well as promote sensitization processes that embrace contextualized interventions, through integrated training [42]. These partnerships enunciate the importance of collaboration to promote more sustainable actions and interventions.

Focusing on equity implementation problems and learning across settings can help achieve the goal of reaching all people [48]. It is sacrosanct to account for diverse specific population groups influenced by gender, political, economic, and other context-specific axes of injustice. The realities of these many groups, many of whom are not even considered stakeholders in NTD efforts, promote their exclusion from health programs. Drawing on the WHO People-Centered Care Framework [49], there is a clear need to focus on equity in health, including precision and justice in health intervention delivery, which should be people-centred, inclusive, holistic, participatory, and responsive to various contexts and specificities. Enabling prevention, cure, and rehabilitation for those at risk of or suffering from NTDs will increase people's potential and prospects while restoring dignity and self-realization and safeguarding the right to health, as stated in the international human rights framework [38]. Social determinants of health play an essential role in the epidemiology of NTDs. The Commission on Social Determinants of Health (CSDH) Framework by the WHO highlights domains in which actions can be targeted to reduce health inequalities contributing to NTDs burden in Africa [50].

In NTD programs, gender equity and mainstreaming should be prioritized. NTD program planners must also include enough time for sensitization and space to collaborate on educational messaging that addresses women's concerns about fertility and the harm it causes to unborn children. Collaborative, multisectoral efforts, including with health professionals working in sexual and reproductive health and maternity and child health, will be crucial in providing advice and education to girls, especially for female Schistosomiasis. In addition to disease-specific targets, the SDGs aim to reduce health disparities and expand access to healthcare in general. If the SDG targets are met, controlling and eliminating NTDs will be easy. Thus, the perpetual state's commitment should collectively engage in actionable goals and improvements. Empowered NTDs control programs and active engagement and synergized efforts of decision-makers, CSOs, research scientists, funding partners, and communities will serve as a good measure of these improvements.

Real-time data-driven decision-making and relevant approaches to NTDs control policies, financing, case management, and community-based intervention should be practised. Africa should harness the data and technology-driven innovation driving the global community in developing and implementing interventions for sustainable change. This necessitates a commitment to strengthening health-care-based data collection and the optimization of NTDs research structure and data management. Innovative research and advocacy tools such as NTDs scorecards are important to ensure no one is left behind. As the world strives to achieve universal health coverage by 2030, it is time for increased continental efforts to combat NTDs. The pandemic of COVID-19 has highlighted the importance of worldwide solidarity in the face of health catastrophes and the urgent need to strengthen health systems. To this, assuring health equity can also help a lot through more investment in health, in keeping with the Abuja Declaration of 2001s goal of spending 15% of the annual budget on health.

## Conclusions

NTDs represent a significant health challenge requiring more focus in the African region. They represent diseases of high prevalence, especially in Sub-Saharan Africa. This high prevalence is also linked to health inequity, with marginalized populations having disproportionate health outcomes. Factors such as poverty of the affected population and continued neglect, especially in health systems funding for disease management, prove crucial. With socioeconomic factors identified as an important determinant for health outcomes, health equity is recognized as a priority for efficient public healthcare delivery. Equity should be adopted in the availability and accessibility of NTDs' preventive and management services. Sustainable interventions with adequate multisector collaboration are required for equity in implementation and coverage.

## Data Availability

The dataset supporting this article's conclusions are included.

## Abbreviations

DALY: Disability-adjusted life years
ESPEN: Expanded special project for the elimination of neglected tropical diseases
NTDs: Neglected tropical diseases
SDGs: Sustainable development goals
WHO: World Health Organization
